# Comparison of Retzius-Sparing Robot-Assisted Radical Prostatectomy vs. Conventional Robot-Assisted Radical Prostatectomy: An Up-to-Date Meta-Analysis

**DOI:** 10.3389/fsurg.2021.738421

**Published:** 2021-09-30

**Authors:** Jiang-Nan Xu, Zhen-Yu Xu, Hu-Ming Yin

**Affiliations:** ^1^Department of Urology, The First Affiliated Hospital of Soochow University, Suzhou, China; ^2^Department of Urology, Kunshan Chinese Medicine Hospital Affiliated to Nanjing University of Chinese Medicine, Suzhou, China

**Keywords:** prostate cancer, Retzius sparing, robot-assisted radical prostatectomy, urinary continence, systematic review and meta-analysis

## Abstract

**Background:** The Retzius space-sparing robot-assisted radical prostatectomy (RS-RARP) has shown better results in urinary continence, but its efficacy and safety compared to conventional robot-assisted radical prostatectomy (c-RARP) remain controversial.

**Material and Methods:** A research was conducted in Medline *via* PubMed, Cochrane Library, EMBASE, and Web of Science up to January 4, 2021, to identify studies comparing RS-RARP to c-RARP. We used RevMan 5.3 and STATA 14.0 for meta-analysis.

**Results:** A total of 14 studies involving 3,129 participants were included. Meta-analysis showed no significant difference in positive surgical margins (PSMs), but the RS-RARP group had significantly higher PSM rates in the anterior site [odds ratio (OR) = 2.25, 95% CI: 1.22–4.16, *P* = 0.01]. Postoperative continence in RS-RARP group at 1 month (OR = 5.72, 95% CI: 3.56–9.19, *P* < 0.01), 3 months (OR = 6.44, 95% CI: 4.50–9.22, *P* < 0.01), 6 months (OR = 8.68, 95% CI: 4.01–18.82, *P* < 0.01), and 12 months (OR = 2.37, 95% CI: 1.20–4.70, *P* = 0.01) was significantly better than that in the c-RARP group. In addition, the RS-RARP group had a shorter console time (mean difference = −16.28, 95% CI: −27.04 to −5.53, *P* = 0.003) and a lower incidence of hernia (OR = 0.35, 95% CI: 0.19–0.67, *P* = 0.001). However, there were no significant differences in estimated blood loss, pelvic lymph node dissection rate, postoperative complications, 1-year-biochemical recurrence rate, and postoperative sexual function.

**Conclusions:** Compared with c-RARP, RS-RARP showed better recovery of continence, shorter console time, and lower incidence of hernia. Although there was no significant difference in overall PSM, we suggest that the surgeon should be more careful if the lesion is in the anterior prostate.

## Introduction

Prostate cancer is the most common malignant tumor in men. The American Cancer Society estimates that there will be 1,919,930 new cases of prostate cancer and 33,330 cancer-related deaths in 2020 (1). In patients with clinically localized prostate cancer, treatment is determined based on risk stratification and life expectancy, including active surveillance, radical prostatectomy, whole gland ablation, and external beam radiation therapy (2). Radical prostatectomy plays an important role in reducing mortality and increasing longevity in patients with clinically localized prostate cancer (3).

In recent years, robot-assisted radical prostatectomy (RARP) has been widely used because of its fine operation in the limited retropubic space. Conventional RARP (c-RARP) was first introduced by Abbou (4) and modified by Menon (5), which is characterized by dissecting the Retzius space to incise and mobilize the bladder and prostate. Despite the good operational advantages of c-RARP, there are some possible adverse consequences, such as urinary incontinence and erectile dysfunction. Among them, urinary incontinence is one of the most serious complications after c-RARP. More than 50% of patients suffer from urinary incontinence at 1 month following radical prostatectomy, which seriously affects the postoperative quality of life (6). With a growing understanding of the anatomy of the prostate and its surrounding structures, many surgical modifications have been proposed in an attempt to improve postoperative functional outcomes while ensuring satisfactory oncological outcomes (7).

Galfano et al. (8) first reported in 2010 that Retzius space-sparing (RS) during RARP was effective in achieving good urinary continence rates. In their subsequent prospective, uncontrolled case series, more than 90% of the 200 patients treated with Retzius space-sparing robot-assisted radical prostatectomy (RS-RARP) achieved immediate continence (9). This surgical approach is characterized by passing through the rectovesical pouch instead of the Retzius space, thus preserving the arcus tendinous, endopelvic fascia, neurovascular bundle, puboprostatic ligament, and deep dorsal vein plexus, which are key structures for maintaining normal urinary continence (10). The efficacy of RS-RARP in urinary continence was also verified in several subsequent studies (11–17).

Despite the better outcomes in urinary continence, several studies have shown that RS-RARP has a higher positive rate of a surgical margin than c-RARP (11–13, 15, 17). However, a recent meta-analysis found the opposite (18). Due to the small sample size of the previous studies and the few references included in the previous meta-analyses, the safety and efficacy of RS-RARP compared with c-RARP are not clear at present. Several new studies have been published in 2020, which may yield new results and new outcome indicators (19–24). Our study aims to systematically compare the clinical, oncological, and functional outcomes of RS-RARP and c-RARP through meta-analysis, to obtain reliable results and provide a basis for future studies and clinical guidance.

## Methods

### Search Strategy

Two researchers independently conducted systematic retrieval of PubMed, EMBASE, Cochrane, and Web of Science, and the retrieval time was up to January 4, 2021. The search terms used include (“Retzius” OR “Bocciardi”) and (“robot” OR “robotic”) and “prostate.” We also browsed references of key articles and manually searched the gray literature to make sure no relevant articles were omitted. Our research was conducted according to the preferred reporting items for systematic reviews and meta-analyses (PRISMA) (25).

### Inclusion and Exclusion Criteria

Inclusion criteria were the following: (a) the subjects were patients with clinically localized prostate cancer; (b) the types of studies were randomized controlled trials (RCTs) or observational controlled studies; (c) studies that involved the comparison of RS-RARP and c-RARP; (d) include at least one of the following outcomes: console time, estimated blood loss, pelvic lymph node dissection (PLND), positive surgical margins, location of positive margins, postoperative continence, complications, hernia, and 1-year-biochemical recurrence rate.

Exclusion criteria were the following: (a) the study was designed as a single-arm trial without a control group; (b) there were no relevant outcome indicators; (c) conference abstracts, case reports, comments, and republished literature; (d) insufficient data or unable to obtain the required data.

### Selection Process and Data Abstraction

The two authors first scanned the titles and abstracts for preliminary screening of all relevant literature. Works of literature that initially meet the inclusion and exclusion criteria or that are controversial will be directly included in the full-text assessment to make sure that all relevant studies are not missed. At the full-text evaluation stage, disputes are negotiated by two authors, and if an agreement cannot be reached, a third author is consulted.

The authors used a predesigned data extraction table to independently extract baseline data and data required for meta-analysis. Baseline data included the following: first author and year of publication, country, study type, mean age, the number of cases, follow-up, outcomes, and quality scores. Outcome indicators included in our study are as follows: console time, estimated blood loss, PLND, positive surgical margins, location of positive margins, postoperative continence, complications, hernia, 1-year-biochemical recurrence rate, and sexual function.

### Literature Quality and Risk of Bias Assessment

To assess literature quality and risk of bias, we evaluated RCTs using the Jadad score (26) and evaluated observational controlled studies using the Newcastle–Ottawa Scale (NOS) (27). In this study, RCTs with a Jadad score of ≥4 were considered to be of high quality, and observational studies with a NOS score of ≥7 were also considered of high quality (26, 27).

### Statistical Analysis

All statistical analyses in our study were performed using RevMan 5.3 (China Cochrane Centre, China; 2014) and Stata (StataCorp, College Station, TX, USA) software, and the significance level was *P* < 0.05. We estimated the effect size of continuous variables by the mean difference (MD) and its 95% CI and estimated the effect size of binary variables by the odds ratio (OR) of the calculated results and its 95% CI. We used inconsistencies (*I*^2^) statistics to assess heterogeneity. If *I*^2^ > 50%, the heterogeneity is very significant and the random-effects model should be adopted. If *I*^2^ < 50%, it indicates that the heterogeneity is acceptable, and a fixed-effect model should be adopted. Subgroup analysis was conducted according to study type and population.

### Sensitivity Analysis and Publication Bias

Sensitivity analysis was conducted by eliminating each literature article one by one, we calculated the change of *I*^2^ through RevMan 5.3 (China Cochrane Centre, China; 2014) and obtained the forest plot of sensitivity analysis through Stata 14. After discovering the source of heterogeneity, we will make a detailed analysis of the target literature to find out the intrinsic reasons for it to be the source of heterogeneity.

Publication bias was assessed quantitatively by Egger's test. When the *p*-value *is* > 0.05, it means there is no significant publication bias. If *P* < 0.05, it indicated the existence of publication bias. In this case, the rim and fill method will be used to assess the impact of publication bias on our results. If publication bias is found to have a significant effect on results, we will discuss it in our discussion.

## Results

### Literature Retrieval Results and Basic Characteristics

**Figure 1:**
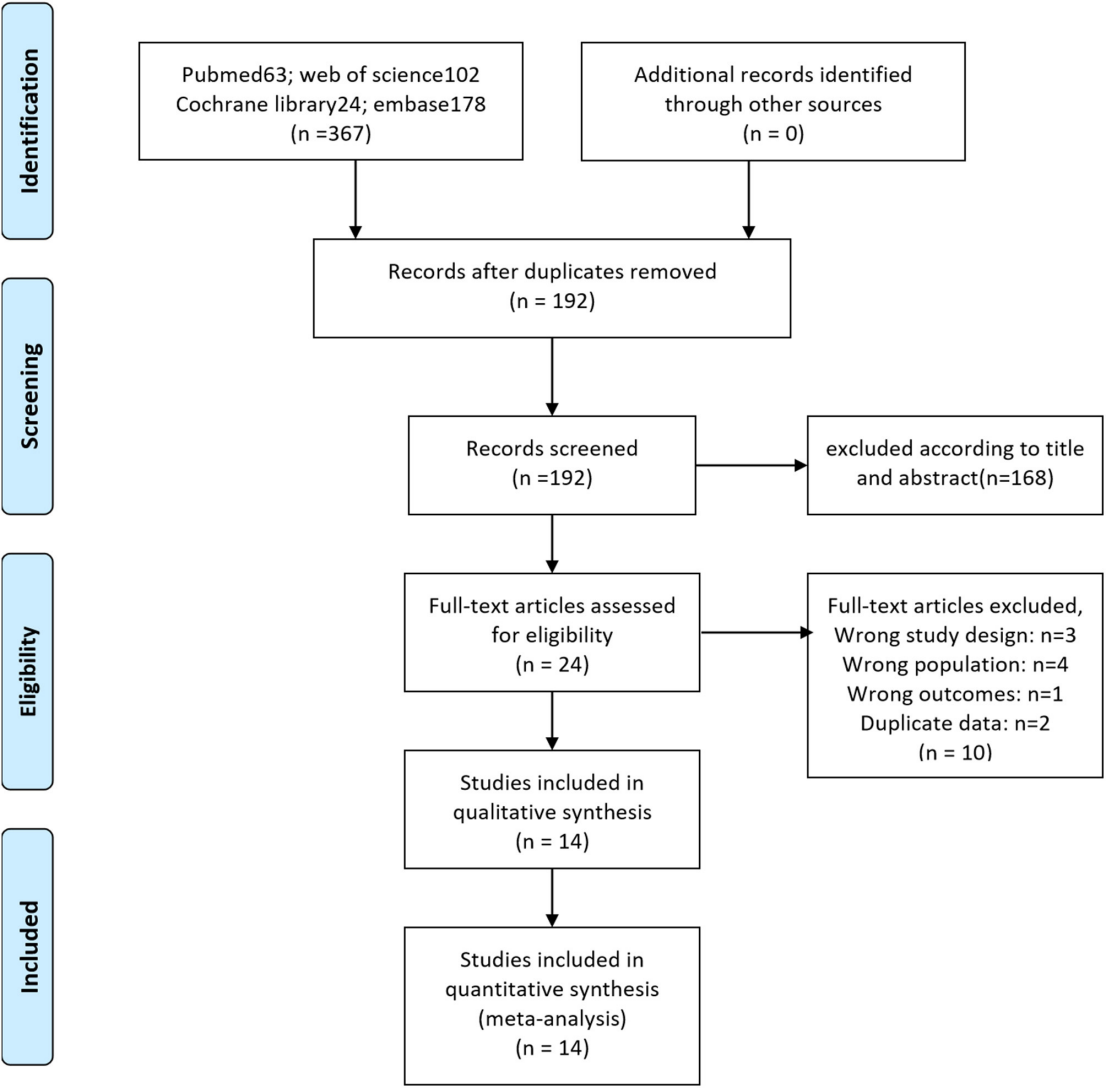
Literature search and selection.

We searched the literature, carefully scanned and screened them, and the specific process is shown in Figure 1. According to the established retrieval formula, we searched a total of 367 related studies, deleted duplicates, and made preliminary screening according to titles and abstracts, and the remaining 24 pieces of literature entered the full-text reading stage. After reading through the full text of 24 articles, a total of 14 studies including 3,129 participants were finally included in our meta-analysis (11–17, 19–24, 28). Of the 14 studies, four were RCTs (11, 12, 16, 20) and the rest were observational controlled studies (13–15, 17, 19, 21–24, 28). Among them, Dalela (16) and Menon (12) were from the same randomized controlled study, and Egan (24) and Kowalczyk (23) were from the same prospective cohort study. The baseline data of the studies included in our meta-analysis are shown in Table 1.

**Table 1:** Literature basic information and literature quality evaluation results.

**Study**	**Country**	**Study type**	**Mean age**		**Sample size**		**Follow-up**	**Outcomes**	**Quality scores**	
			**RS**	**Non-RS**	**RS**	**Non-RS**		**(mon)**	**Jadad**	**NOS**
Lim (17)	Korea	PPSM	65.7	66.2	50	50	6	ABCDEF	NA	9
Chang (28)	Korea	CS	65.0	65.0	298	541	24	G	NA	8
Dalela (16)	USA	RCT	61.0	61.5	60	60	12	ACDEFH	4	NA
Menon (12)										
Sayyid (15)	USA	PS	61.0	62.0	100	100	12	ACDEF	NA	8
Chang (14)	China	PPSM	64.4	67.5	30	30	12	BDEH	NA	9
Eden (13)	UK	PS	63.0	65.0	40	40	3	BDEFI	NA	7
Asimakopoulos (11)	Italy	RCT	66.0	65.0	39	40	6	CDEF	4	NA
Egan (24)	USA	PS	62.1	61.9	70	70	12	ABDEFGH	NA	9
Kowalczyk (23)										
Lee (22)	Korea	PPSM	65.0	66.0	609	609	6	BDEFI	NA	8
Liao (21)	China	RC	64.8	65.6	41	92	12	BDEH	NA	7
Ota (20)	Japan	RC	67.0	69	25	25	12	ABDEFG	NA	8
Qiu (19)	China	RCT	68.0	67.0	55	55	12	ABCDEF	4	NA

*A: console time; B: estimated blood loss; C: pelvic lymph node dissection; D: positive surgical margins; E: postoperative continence; F: complications; G: hernia; H: 1-year-biochemical recurrence rate; I: Sexual function RS: Retzius-sparing; PPSM: Prospective propensity score matching; CS: case-control; RCT: Randomized controlled trial; PS: Prospective study; RC: Retrospective cohort*.

### Methodological Quality Assessment

We evaluated RCTs using the Jadad score (26) and evaluated observational controlled studies using the NOS (27). After detailed evaluation according to the scoring protocol, we found that all RCTs had a Jadad score greater than or equal to 4, and all observational studies had a NOS score greater than or equal to 7, indicating that all included studies had good methodological quality (Table 1).

### Meta-Analysis Results

#### Console Time

**Figure 2:**
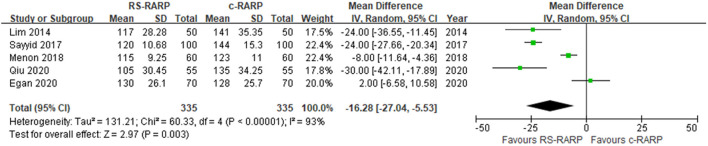
Forest plot of console time.

Five studies (12, 15, 17, 19, 24) reported the difference in console time between RS-RARP and c-RARP. Due to the high heterogeneity (*I*^2^ = 93%), the meta-analysis results using the random-effects model showed that the console time of RS-RARP was significantly shorter than that of c-RARP (MD = −16.28, 95% CI: −27.04 to −5.53, *P* = 0.003) (Figure 2).

#### Estimated Blood Loss

**Figure 3:**
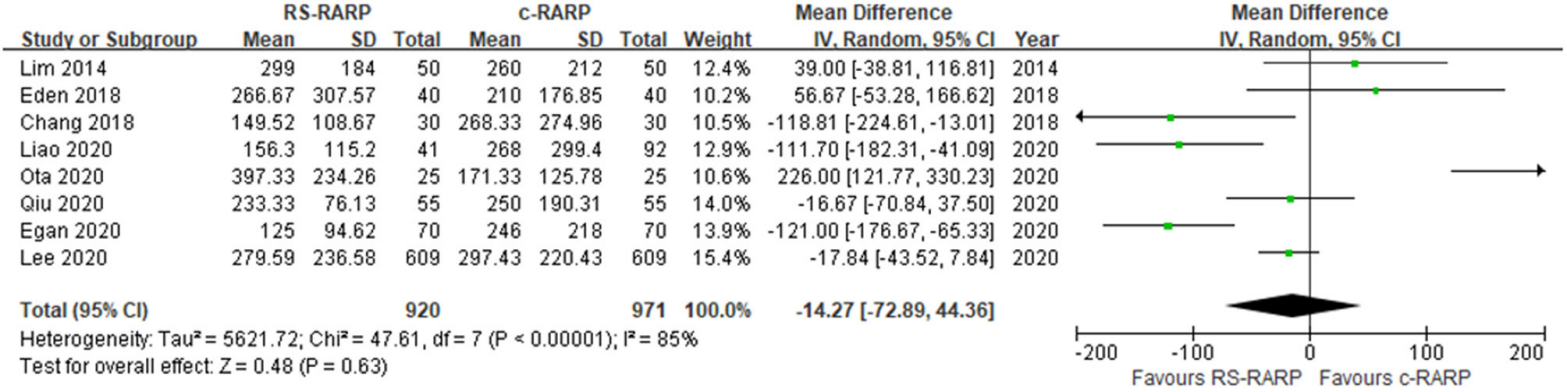
Forest plot of estimated blood loss.

Eight studies (13, 14, 17, 19–22, 24) reported the difference in estimated blood loss between RS-RARP and c-RARP. Due to the high heterogeneity (*I*^2^ = 85%), the meta-analysis results using the random-effects model showed that there was no significant difference in estimated blood loss between RS-RARP and c-RARP (MD = −14.27, 95% CI: −72.89 to 44.36, *P* = 0.63) (Figure 3).

#### Pelvic Lymph Node Dissection

**Figure 4:**
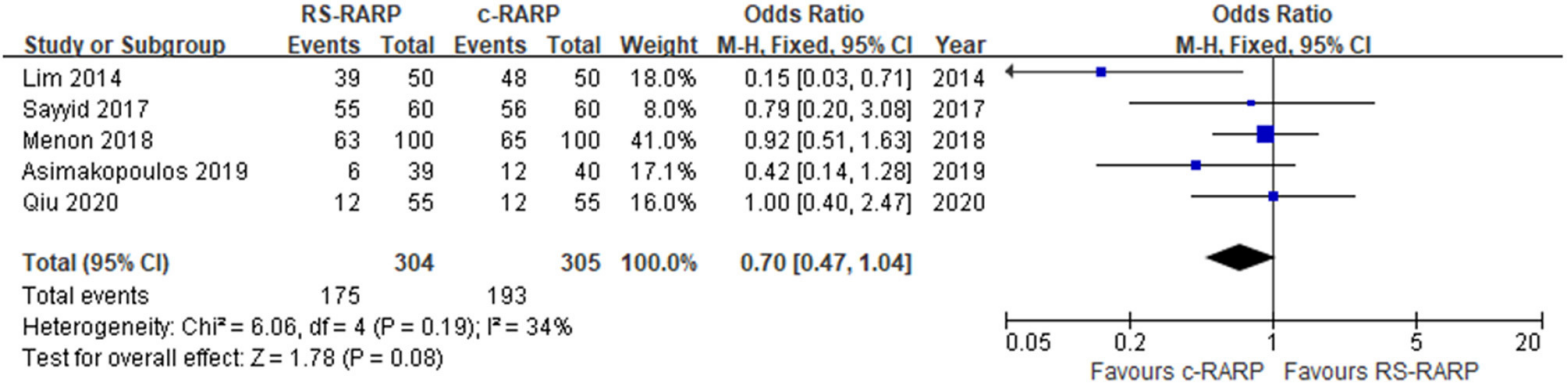
Forest plot of pelvic lymph node dissection (PLND).

Five studies (11, 12, 15, 17, 19), including 609 participants, reported PLND rate. Meta-analysis using a fixed-effects model showed that there was no significant difference in PLND rate between the RS-RARP group and the c-RARP group (OR = 0.7, 95% CI: 0.47–1.04, *P* = 0.08). *I*^2^ = 34%, the heterogeneity was in the acceptable range (Figure 4).

#### Positive Surgical Margins

**Figure 5:**
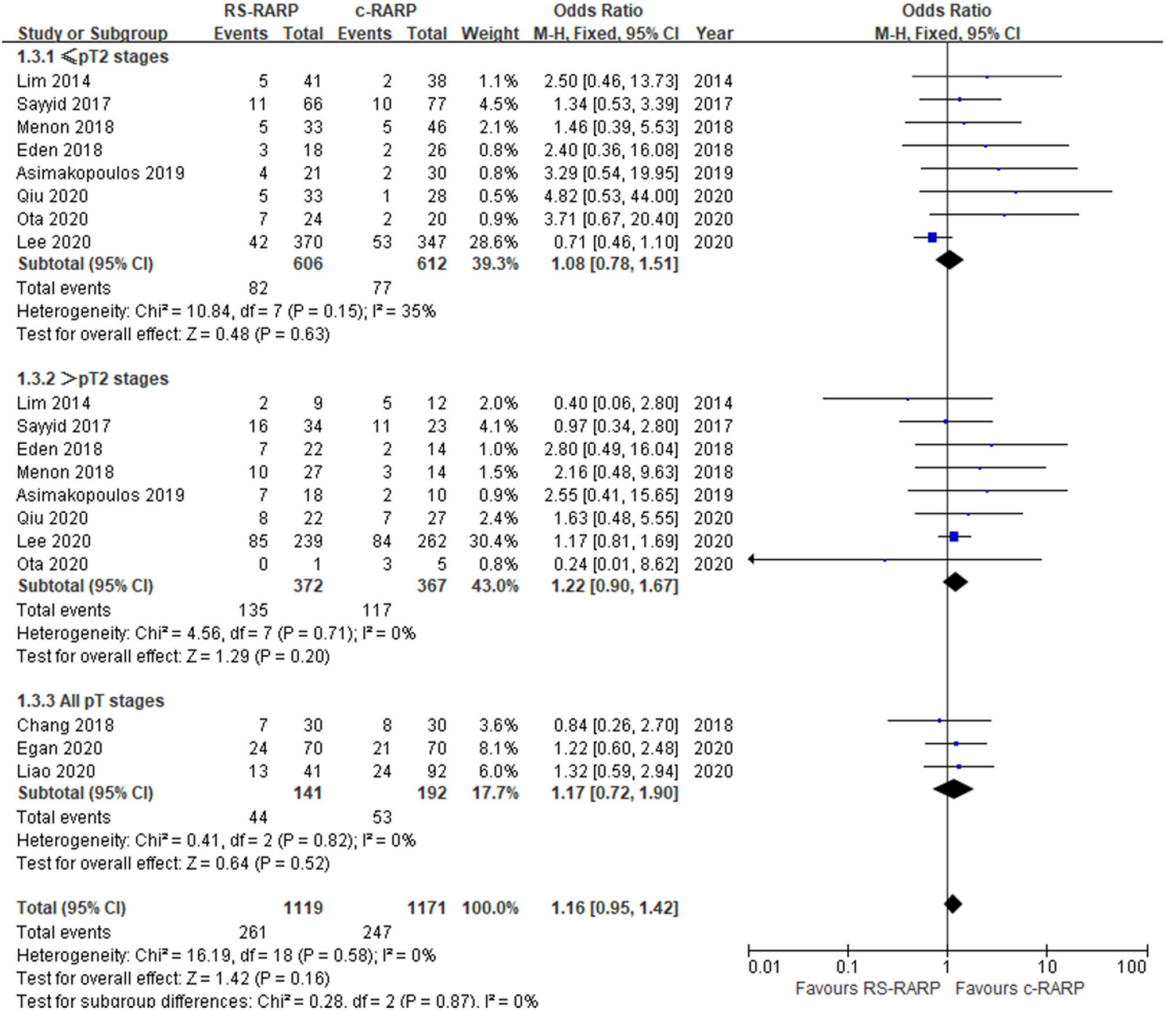
Forest plot of positive surgical margins.

PSM data were reported in 11 studies (11–15, 17, 19–22, 24) involving a total of 2,290 participants. Our meta-analysis showed that there was no significant difference in PSM rates between RS-RARP and c-RARP (OR = 1.16, 95% CI: 0.95–1.42, *P* = 0.16). *I*^2^ = 0, no obvious heterogeneity was observed. In the subgroup based on pathological stage, we found that no matter if pathological stage ≤ pT2 (OR = 1.08, 95% CI: 0.78–1.51, *P* = 0.63) or > pT2 (OR = 1.22, 95% CI: 0.90–1.67, *P* = 0.20), there was no significant difference in PSM rates between the two surgical methods (Figure 5).

**Figure 6:**
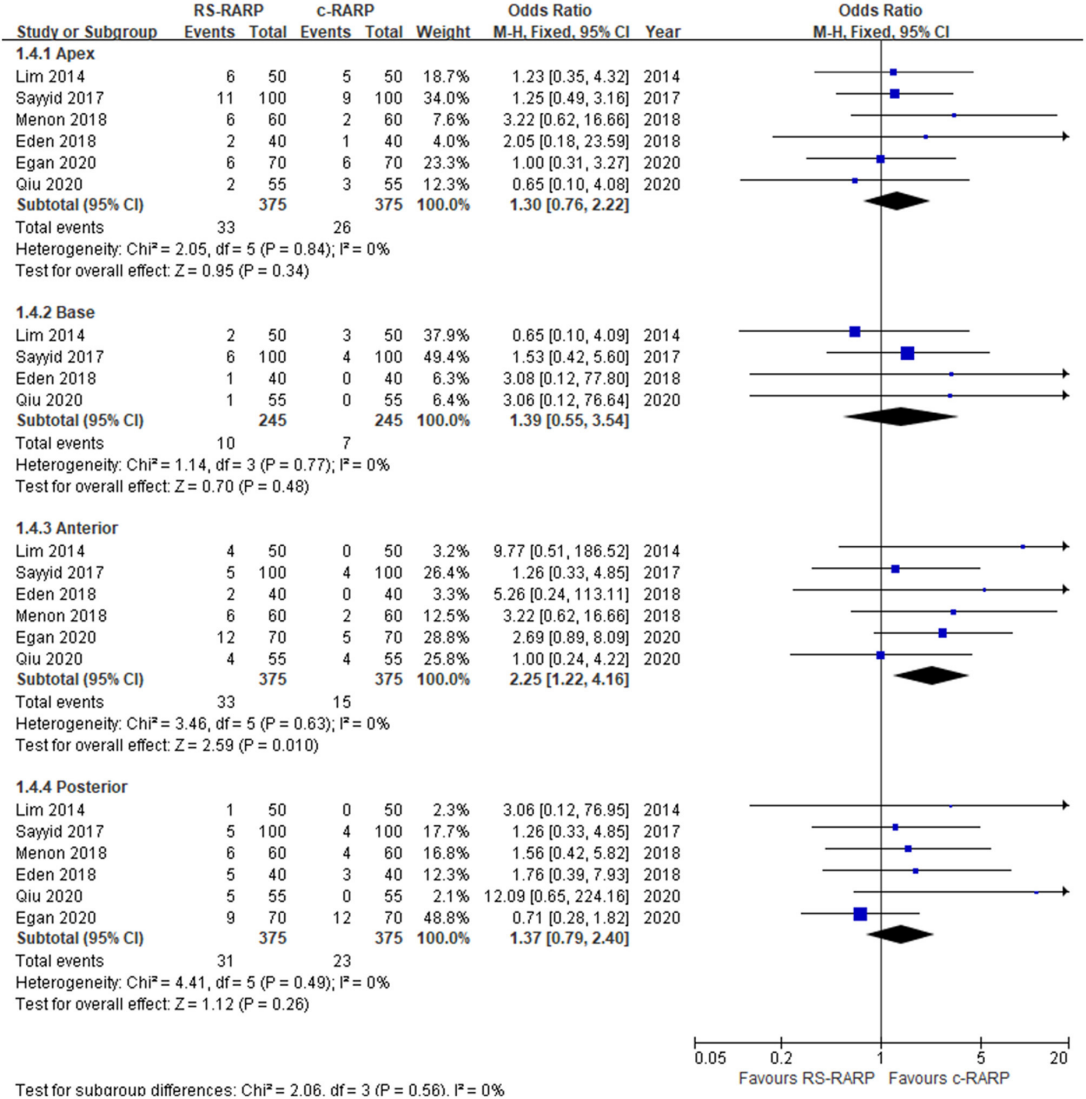
Forest plot of location of positive margins.

We also conducted in-depth analysis according to the location of positive margins. Six studies (12, 13, 15, 17, 19, 24) reported data on the location of positive surgical margins, and we found that compared with c-RARP, RS-RARP had significantly higher PSM rates in the anterior site (OR = 2.25, 95% CI: 1.22–4.16, *P* = 0.01). In the other three sites, including apex (OR = 1.30, 95% CI: 0.76–2.22, *P* = 0.34), base (OR = 1.39, 95% CI: 0.55–3.54, *P* = 0.48), and posterior (OR = 1.37, 95% CI: 0.79–2.40, *P* = 0.26), there was no significant difference in PSM rates between the two surgical methods (Figure 6).

#### Postoperative Continence

**Figure 7:**
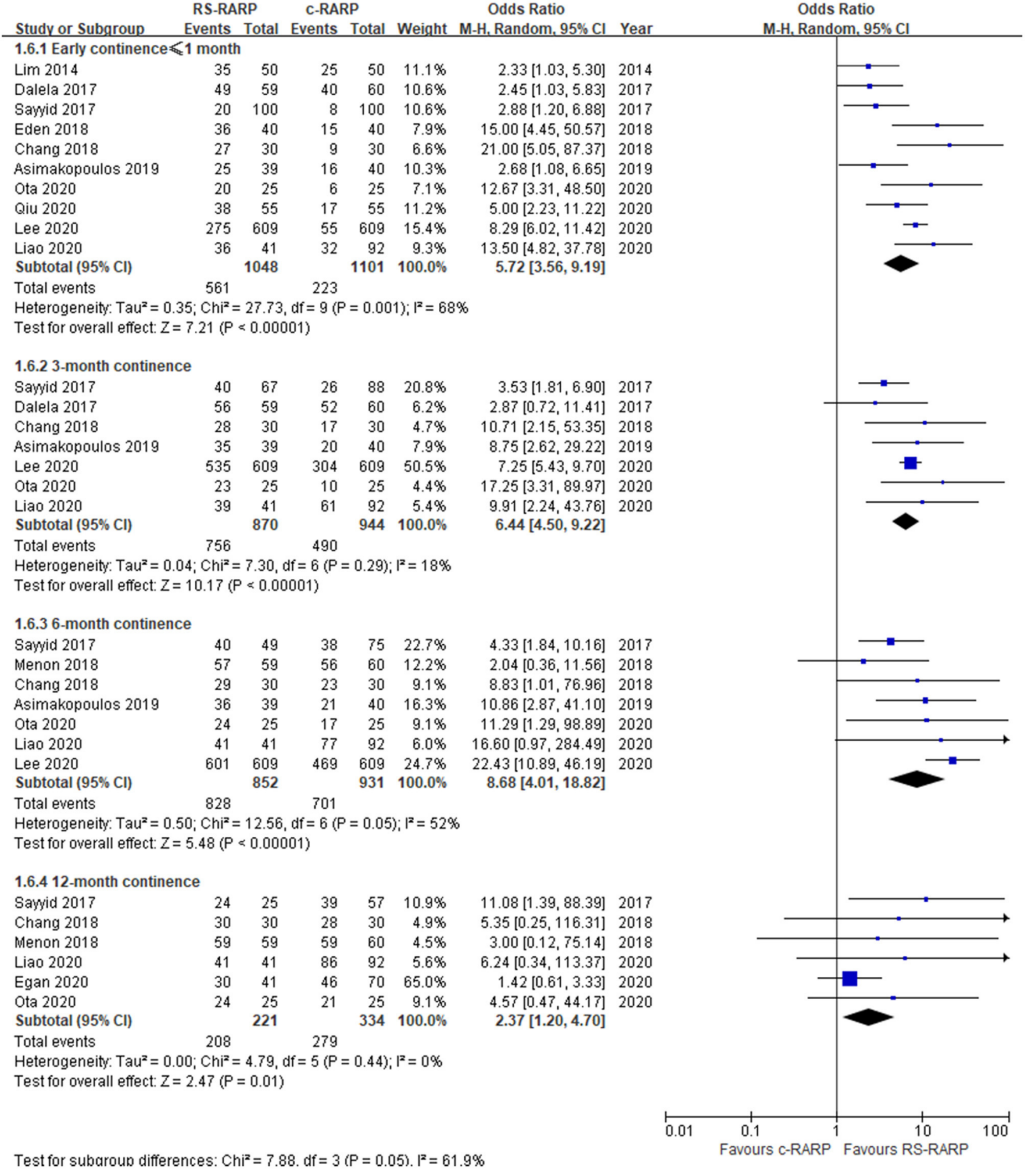
Forest plot of postoperative continence.

Ten studies (11, 13–17, 19–22) reported data on early urine continence ( ≤ 1 month), and the random-effects model results showed that RS-RARP was significantly better than c-RARP in early urine continence (OR = 5.72, 95% CI: 3.56–9.19, *P* < 0.001, *I*^2^ = 68%) (Figure 7).

Seven studies (11, 14–16, 20–22) reported data on 3-month continence, and the results of the fixed-effect model showed that RS-RARP was significantly better than c-RARP in 3-month continence (OR = 6.44, 95% CI: 4.50–9.22, *P* < 0.001, *I*^2^ = 18%) (Figure 7).

Seven studies (11, 12, 14, 15, 20–22) reported data on 6-month continence, and the random-effect model results showed that RS-RARP was significantly better than c-RARP in 6-month continence (OR = 8.68, 95% CI: 4.01–18.82, *P* < 0.001, *I*^2^ = 52%) (Figure 7).

Six studies (12, 14, 15, 20, 21, 24) reported data on 12-month continence, and the fixed-effects model results showed that RS-RARP was significantly better than c-RARP in 12-month continence (OR = 2.37, 95% CI: 1.20–4.07, *P* = 0.01, *I*^2^ = 0%) (Figure 7).

#### Complications and Hernia

**Figure 8:**
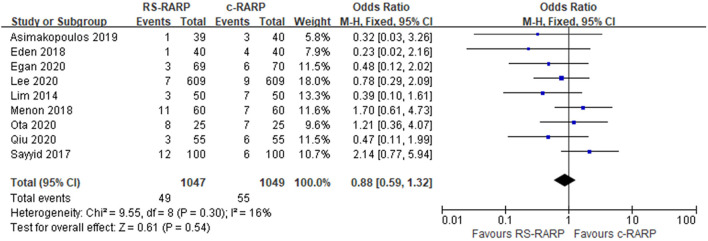
Forest plot of complications.

**Figure 9:**
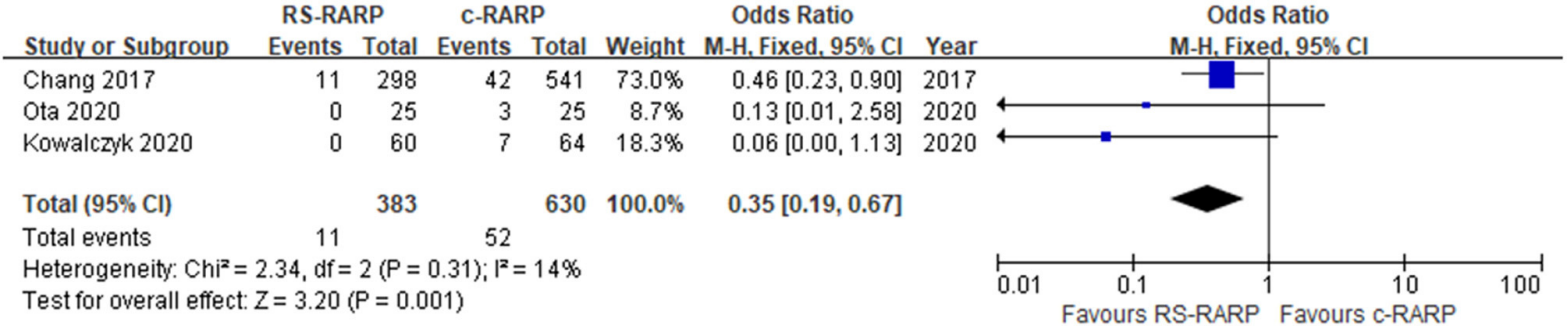
Forest plot of hernia.

A total of nine studies (11–13, 15, 17, 19, 20, 22, 24) reported postoperative complications, and three studies (20, 23, 28) reported postoperative hernia incidence. Results of the meta-analysis showed that although there was no significant difference in postoperative complications between the two surgical procedures (OR = 0.88, 95% CI: 0.59–1.32, *P* = 0.54, *I*^2^ = 16%) (Figure 8), the incidence of postoperative hernia in the RS-RARP group was significantly lower than that in the c-RARP group (OR = 0.35, 95% CI: 0.19–0.67, *P* = 0.001, *I*^2^ = 14%) (Figure 9).

#### 1-Year-Biochemical Recurrence Rate

**Figure 10:**
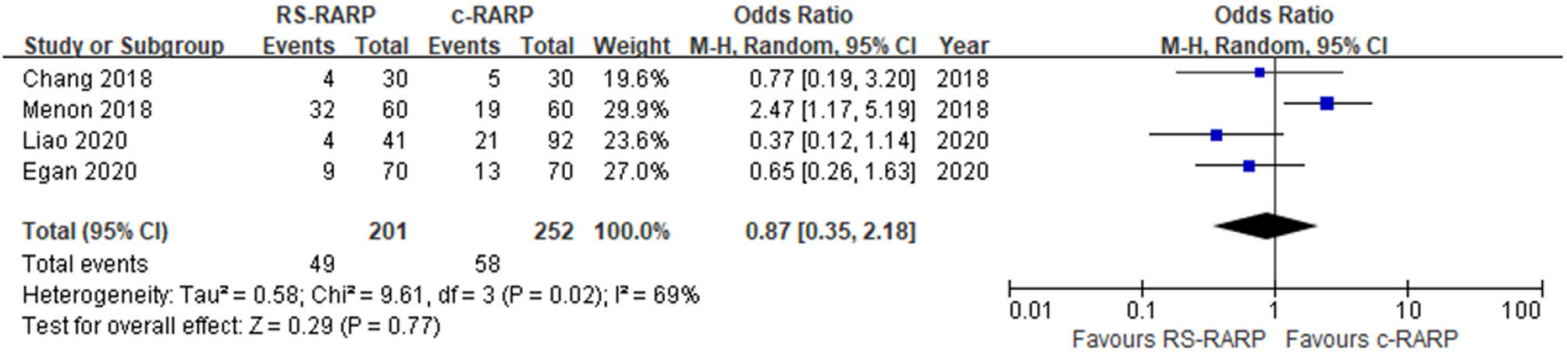
Forest plot and of 1-year-biochemical recurrence rate.

Biochemical recurrence data were reported in four studies (12, 21, 24, 28), and meta-analysis using a random-effects model showed no significant difference in the rate of 1-year-biochemical recurrence between the two surgical procedures (OR = 0.87, 95% CI: 0.35–2.18, *P* = 0.77, *I*^2^ = 69%) (Figure 10).

#### Postoperative Sexual Function

The study of Egan et al. (24) [expanded prostate cancer index composite for clinical practice (EPIC-CP) sexual function scores: 4.6 ± 3.4 vs. 5.3 ± 2.6; *P* = 0.417] and Lee et al. (22) [international index of erectile function-5 scores (IIEF-5) score: 13 ± 7.2 vs. 13 ± 7.4; *P* = 0.9] showed no significant difference in postoperative sexual function between the two surgical methods.

### Subgroup Analysis

We performed the subgroup analyses of functional and oncological outcomes by study type and population. As shown in Table 2, the results of the observational study subgroup were consistent with the overall results of our meta-analysis, while in the RCT subgroup, the RS-RARP group seemed to have a higher margin positive rate and biochemical recurrence rate than the c-RARP group. In population-based subgroup analysis, we found that the advantage of RS-RARP in urine continence appeared to be more pronounced in the Asian population. In addition, in the western population subgroup, the positive rate of surgical margin in the RS-RARP group still seemed to be higher than that in the C-RARP group. Specific subgroup analysis results are shown in Table 2.

**Table 2:** Subgroup analysis.

**Subgroup analysis**	**Positive surgical margins (≤pT2)**	**Positive surgical margins (>pT2)**	**Positive surgical margins (All)**	**Early-continence (≤1month)**	**12-month continence**	**1-year-biochemical recurrence rate**
Study type						
RCT	2.42 [0.95, 6.16]	1.97 [0.86, 4.55]	2.16 [1.16, 4.02]	3.29 [2.00, 5.40]	3.00 [0.12, 75.14]	2.47 [1.17, 5.19]
Observational	0.96 [0.67, 1.37]	1.17 [0.83, 1.64]	1.17 [0.72, 1.90]	7.50 [4.21, 13.38]	2.91 [1.19, 7.11]	0.56 [0.29, 1.06]
Population						
Asian	0.98 [0.69, 1.41]	1.14 [0.81, 1.61]	1.05 [0.83, 1.34]	7.41 [4.26, 12.89]	5.19 [1.11, 24.36]	0.49 [0.20, 1.19]
western	2.06 [0.82, 5.18]	1.64 [0.82, 3.29]	1.50 [1.01, 2.23]	3.73 [1.83, 7.61]	2.82 [0.69, 11.49]	1.30 [0.35, 4.82]

*RCT, Randomized controlled trial*.

### Sensitivity Analysis

**Figure 11:**
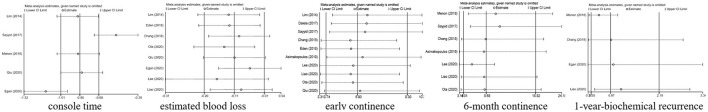
Sensitivity analysis.

In the meta-analysis of console time, blood loss, early continence, 6-month continence, and 1-year-biochemical recurrence rate, we found significant heterogeneity (93, 85, 68, 52, and 69%, respectively). We performed a sensitivity analysis using the Stata software and produced forest plots after each study was sequentially removed. As shown in Figure 11, we found that in the outcome index group of console time, Sayyid (15) and Egan (24) may be sources of heterogeneity. In the remaining four outcome indicator groups, the combined effect value after each study was successively removed and was between the two reference lines. At the same time, when we changed the random-effects model to the fixed-effects model, the results of the meta-analysis did not significantly change. It can be seen that in the remaining four outcome indicator groups, although heterogeneity existed and sensitivity analysis did not find a clear source of heterogeneity, it did not bring significant bias to our results, and our results were still stable.

### Publication Bias

**Figure 12:**
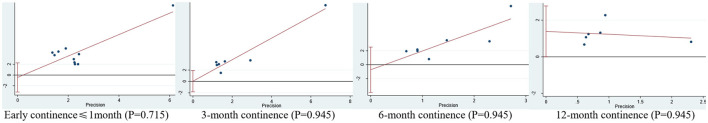
Publication bias.

We quantitatively evaluated publication bias by Egger's test, and the results showed that no obvious publication bias was found in all the outcome indicator groups. We showed the Egger graph and the *p*-value of some major outcome indicators in Figure 12.

## Discussion

To our knowledge, our meta-analysis is the most up-to-date and comprehensive. Due to the inclusion of several recent high-quality works of literature (19–24), we have obtained some more stable results in some outcome indicators that were different from the previous meta-analyses (18, 29, 30). In addition, we are the first study to include the PLND rate in the meta-analysis, and also the first study to conduct the subgroup analysis based on the population. Meta-analysis showed no significant difference in PSM, but the RS-RARP group had significantly higher PSM rates in the anterior site. The postoperative continence rate of the RS group at 1, 3, 6, and 12 months was significantly higher than that of the c-RARP group. In addition, the RS-RARP group had a shorter console time and a lower incidence of hernia. However, there were no significant differences in estimated blood loss, PLND rate, postoperative complications, and 1-year-biochemical recurrence rate. Our subgroup analysis found that RS-RARP seemed to have a higher margin positive rate in the RCT subgroup. In the subgroup analysis by population, we found that the advantage of RS-RARP in urine continence appeared to be more pronounced in the Asian population.

RS-RARP can be called “reverse perineal or RP” in a sense because it combines the advantages of perineal RP and retropubic RP (17). Perineal RP can accurately dissect the urethra and preserve the Retzius space and dorsal venous complex (DVC), but it damages the pelvic floor muscles and can lead to severe urinary incontinence (31). In contrast, the retropubic RP avoids damage to the pelvic floor muscles, but requires dissection of the Retzius space, resulting in the injury of critical structures involved in urine continence, such as arcus tendineus, endopelvic fascia, and neurovascular bundle (31). RS-RARP preserves both Retzius space and pelvic floor muscles, minimizes surgical trauma, and retains normal anatomical structure to the greatest extent. Although c-RARP also includes several remedial steps that have been shown to improve postoperative urine continence, such as the posterior reconstruction of the rhabdosphincter (32), bladder neck ultradissection (33), puboperineoplasty (34), and nerve-sparing dissection (35), postinjury reparation is never as effective as outright injury avoidance. This explains why RS-RARP is significantly better than c-RARP in early urine continence.

In terms of clinical outcomes, we found that the RS-RARP group had shorter console time and a lower incidence of hernia, but no significant differences in estimated blood loss and complications. The difference in operative time may be due to the fact that RS-RARP maximizes the preservation of natural anatomy and does not require remedial reconstruction. As for the difference in hernia incidence, Shimbo et al. (36) noted that urethrovesical anastomosis during c-RARP surgery might lead to overstretching of the peritoneum and vas deferens, resulting in medial displacement and enlargement of the inner ring, leading to increased hernia incidence. Compared with c-RARP, RS-RARP can maximize the protection of the anterior compartment and myopectineal orifice to prevent the displacement of the internal ring, thus greatly reducing the incidence of hernia (17, 37). Although RS-RARP is theoretically less invasive than c-RARP, there is no significant difference in estimated blood loss. This might be due to the fact that during c-RARP, urine spills less from the bladder, but during RS-RARP, urine constantly spills from the bladder neck, which is open above the lens, due to gravity (22). This difference in urine content might bias the estimation of blood loss.

The results of some preliminary studies (11, 13) and meta-analysis (29) suggested that RS-RARP might have a higher PSM rate than c-RARP, while our meta-analysis showed no significant difference in PSM rate between the two surgical methods, which may be due to the learning curve of a new surgical procedure. Galfano et al. (9) reported an incidence of PSM of 32% in the first 100 patients who underwent RS-RARP and 19% in the next 100 patients. Recent studies have shown that the PSM rate of RS-RARP is very low when the operator is experienced (19, 22, 24). Lee et al. (22) based on a large sample found no significant difference in PSM between RS-RARP and c-RARP. The study of Egan et al. (24) also showed the same result. Although there was no significant difference in overall PSM, our subgroup analysis showed that the RS-RARP group had significantly higher PSM rates in the anterior site. In particular, in the study of Egan et al. (24), the PSM rates in the anterior site in the RS-RARP group were 2.69 times that of c-RARP. Despite the high literature quality of the study of Egan et al. (24), to avoid bias, we tried to remove the data of this study and found that although the difference became not statistically significant (OR = 2.07, 95% CI: 0.99–4.36, *P* = 0.05), the clinical trend was still obvious. Lim et al. (17) suggested that part of the reason for PSMs at the anterior margins may be related to anatomy. There is no clear plane between the prostatic stroma and the urethral sphincter muscle fibers at the apex and anterior (38). In addition, Kim et al. (39) believed that surgeons had a certain degree of vision limitation when performing the anterior aspect, which may also be one of the reasons. Our results are also somewhat supported by a recent study showing that patients with transitional zone tumors receiving RS-RARP had a higher rate of PSM, with most PSMs (39.8%) located in the anterior part of the prostatic gland (40). This study also indicates that the anterior part of the prostate capsule is often defective, resulting in a lack of a clear plane between the prostate capsule and the fibromuscular stroma. Therefore, patients with tumors located in the transitional zone, especially in the anterior part, are more likely to develop PSM during RS-RARP, which is characterized by anterior preservation (40). Perhaps, in theory, the RS-RARP approach is more suitable for posterior rather than anterior tumors. Therefore, when facing anterior tumors with higher pathological stages, surgeons can move slightly forward away from the prostate during the operation and remove more periprostatic fat to avoid PSM, or they can also consider choosing c-RARP (17). At present, there is no significant difference in the 1-year-biochemical recurrence rate between the two surgical methods, which is consistent with the results of PSM and reflects the oncologic safety of RS-RARP to a certain extent. Further follow-up is still needed.

Whether PLND is performed or not affects the clinical, functional, and oncological outcomes of patients (17). To avoid bias caused by differences in PLND rates between the groups, we included the PLND rate as one of the outcome indicators in our meta-analysis. Our results showed that there was no significant difference in PLND rate between the groups, which not only confirmed the operability of PLND in RS-RARP but also basically excluded the possibility that PLND rate could bring about bias to the results.

In our subgroup analysis, we found that the Asian population seemed to be more suitable for RS-RARP and had better function and oncological outcomes. This might be due to the fact that most of the studies (19–22) on the Asian populations were published recently, surgeons have gained more experience than earlier studies (12, 13, 15, 16) on the Western populations, and the RS-RARP technique itself also has been improved in many details. Whether this difference is really meaningful is unknown and may require further anatomical studies to confirm.

In our sensitivity analysis, the vast majority of the heterogeneity was not sourced, but despite the heterogeneity, our results were robust. Our heterogeneity mainly existed in the operation time and urine control outcome indicator group. The operation time may be related to the learning curve and recording method, and the definition of urine continence may also have some differences in various medical institutions, which may be the reason for the high heterogeneity.

## Limitations

There are some limitations to our study. First, although we have explored postoperative sexual function, there are few solid results due to the limited data available. Second, there is a lack of long-term survival data. Third, we cannot yet fully explain the differences in outcomes between different populations.

## Conclusion

Compared with c-RARP, RS-RARP showed better recovery of continence, shorter console time, and lower incidence of hernia. Although there was no significant difference in overall PSM, we suggest that the surgeon should be more careful if the lesion is in the anterior prostate.

## Data Availability Statement

The original contributions presented in the study are included in the article/supplementary material, further inquiries can be directed to the corresponding author.

## Ethics Statement

The authors are accountable for all aspects of the work in ensuring that questions related to the accuracy or integrity of any part of the work are appropriately investigated and resolved.

## Author Contributions

H-MY conception, design, and administrative support. J-NX and Z-YX provision of study materials or patients, collection and assembly of data, data analysis, and interpretation. All authors write the manuscript and approval of manuscript.

## Conflict of Interest

The authors declare that the research was conducted in the absence of any commercial or financial relationships that could be construed as a potential conflict of interest.

## Publisher's Note

All claims expressed in this article are solely those of the authors and do not necessarily represent those of their affiliated organizations, or those of the publisher, the editors and the reviewers. Any product that may be evaluated in this article, or claim that may be made by its manufacturer, is not guaranteed or endorsed by the publisher.
